# Media Use During the COVID-19 Pandemic: Cross-sectional Study

**DOI:** 10.2196/33011

**Published:** 2022-06-07

**Authors:** Marjolaine Rivest-Beauregard, Justine Fortin, Connie Guo, Sabrina Cipolletta, Ram P Sapkota, Michelle Lonergan, Alain Brunet

**Affiliations:** 1 Department of Psychiatry McGill University Montréal, QC Canada; 2 Division of Psychosocial Research Douglas Mental Health University Institute Montréal, QC Canada; 3 Department of Psychology University of Québec in Montréal Montréal, QC Canada; 4 Department of Psychology University of Padua Padua Italy; 5 School of Psychology University of Ottawa Ottawa, ON Canada

**Keywords:** media use, support, information-seeking behaviors, trauma- and stressor-related symptoms, COVID-19, media, information-seeking, behavior, trauma, stress, symptom, frequency, risk, distress

## Abstract

**Background:**

Throughout the pandemic, the general population was encouraged to use media to be kept informed about sanitary measures while staying connected with others to obtain social support. However, due to mixed findings in the literature, it is not clear whether media use in such a context would be pathogenic or salutogenic.

**Objective:**

Therefore, the associations between COVID-19–related stressors and frequency of media use for information-seeking on trauma- and stressor-related (TSR) symptoms were examined while also investigating how social media use for support-seeking and peritraumatic distress interact with those variables.

**Methods:**

A path model was tested in a sample of 5913 adults who completed an online survey.

**Results:**

The number of COVID-19–related stressors (β=.25; *P*<.001) and extent of information-seeking through media (β=.24; *P*=.006) were significantly associated with the severity of TSR symptoms in bivariate comparisons. Associations between levels of peritraumatic distress and both COVID-19–related stressors and information-seeking through media, and social media use for support- and information-seeking through media were found (β_COVID-19 stressors: Peritraumatic Distress Inventory_=.49, *P*<.001; β_seeking information: Peritraumatic Distress Inventory_=.70, *P*<.001; β_seeking information–seeking support_=.04, *P*<.001).

**Conclusions:**

Results suggest that exposure to COVID-19–related stressors and seeking COVID-19–related information through the media are associated with higher levels of peritraumatic distress that, in turn, lead to higher levels of TSR symptoms. Although exposure to the stress of the COVID-19 pandemic may be unavoidable, the frequency of COVID-19–related information consumption through various media should be approached with caution.

## Introduction

### The COVID-19 Pandemic, Media Use, and Mental Health

The COVID-19 pandemic has resulted in global destabilization and repeated confinement of billions of people to their homes, and has made *physical distancing* a new way of life. In the early days of the pandemic and for quite some time, governmental authorities have broadcasted bleak news updates through the media daily (eg, [1,2]) and have urged people to stay connected to each other virtually to offset the absence of social contacts (eg, [3]). While some literature suggests that using social media platforms for support is salutogenic (ie, supporting health and well-being) [4,5], others suggest that media consumption involves repeated exposure to aversive content inducing pathogenic effects, including trauma- and stressor-related (TSR) symptoms typically associated with adjustment disorder or posttraumatic stress disorder (PTSD), as per the *Diagnostic and Statistical Manual of Mental Disorders* (Fifth Edition) [6-8].

The reliance on media (eg, social media, television, or news reports) during a global crisis like the COVID-19 pandemic has multifaceted benefits, including the dissemination of urgent information and public health guidelines. In previous infectious disease outbreaks, the provision of information through the media has fostered preventive behaviors in the general population, such as wearing a mask, avoiding crowded public spaces, and handwashing [9]. However, this media presence may also come at the cost of experiencing psychological distress, including depression [10], PTSD symptoms [10-13], and anger [9], particularly if the media disseminate bleak or sensationalistic information or misinformation on a large scale [14], a phenomenon known as an “infodemic” [15,16]. That being said, the silver lining is that social media can also be used for various other purposes, such as seeking social connection and support [17-20], which can be associated with better mental health outcomes, including lower levels of anxiety, depression, and stress [5,18].

Empirical findings on the relation between different forms of media use and mental health have been mixed (eg, [21,22]), highlighting the complex nature of this association. In the context of a mass disaster such as the COVID-19 pandemic [23] and given the stressful and traumatic nature of this event for some [6,24], one must consider an array of trauma-related variables to better understand how media use is associated with TSR outcomes. Indeed, peritraumatic distress is a strong predictor of TSR symptoms [25], which could, in turn, be further exacerbated by media use when exposed to more gory details of the events [26]. Such associations should be taken into consideration when contemplating media use in a stressful or traumatic context.

Additionally, much of the current evidence regarding the pathogenic effects of media use is based on social media such as Facebook (eg, [21,22]). However, media use goes far beyond social media and is a constantly evolving domain. We use the term “media” to refer to traditional and new media. The concept, as used in this paper, includes all classical mass media such as newspapers, magazines, radio, and television, and their online versions (the latter belong to mainstream news media, even if they use an electronic format). “Social media” is used to designate various forms of consumer-generated content such as blogs, social network sites, forums, virtual communities, online newspaper reader comments, and media files shared on sites such as YouTube. Additionally, several studies have distinguished between passive (involves information consumption) and active (involves connectiveness) media use (eg, [27,28]), suggesting that active use is associated with positive outcomes on one’s mental health. However, the literature on passive media use still presents mixed results [27], highlighting the need for more fine-grained research. In this manuscript, *media use* is defined as media engagement for informative purposes and media engagement for connectiveness and support purposes.

The differential susceptibility to media effects model (DSMM) is a framework that ties together media- and non-media–related variables so as to better understand the complexity of the associations between media use and mental health [29]. According to the DSMM [29], many variables, such as social context and individual differences including cognitive, affective, behavioral, and physiological factors, moderate the associations between media use and mental health. For instance, differential-susceptibility variables, whether dispositional (eg, gender, motivations, or values), developmental (eg, cognitive, social, or emotional development), or social (eg, peers, family, or work context), can be moderators of the association with mental health. However, the DSMM also suggests that the association between media use and mental health are indirect and can be mediated by various cognitive and emotional factors [29]. Previous research has used this model to investigate the pathogenic effects of media exposure following other mass disasters such as the 9/11 attacks and the Iraq war [13,30]. More recently, this model has been applied in the context of the COVID-19 pandemic [31], where the authors investigated the mediating role of negative affect on the association between social media use and TSR symptoms; however, the authors did not include a measure of peritraumatic distress, one of the strongest predictors of TSR symptoms [25].

### Proposed Model and Hypotheses

**Figure 1:**
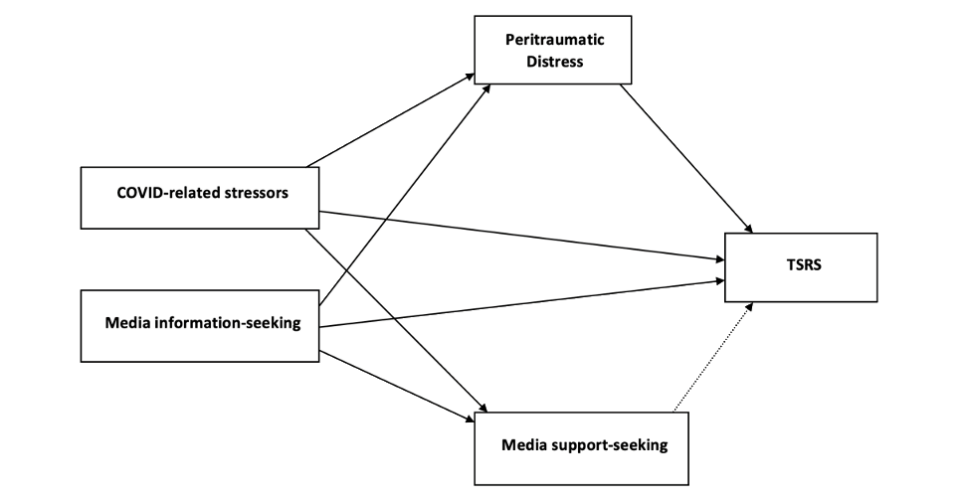
Hypothetical path model. Solid lines represent positive relationships, while the dotted line represents a negative relationship. TSRS: trauma- and stressor-related symptoms.

We operationalized media use as defined by the authors of the DSMM: the broad use of various media types such as social networks, virtual environments, and traditional media (eg, newspapers and televisions) [29]. We also further divided media use according to the intended use (ie, information-seeking behaviors and support-seeking behaviors). Thus, in this research, we aimed to explore the associations between COVID-19–related stressors and media use for information-seeking with TSR symptoms, and the role of media use for support-seeking and peritraumatic distress and this association. We hypothesized that COVID-19–related stressors and using media for COVID-19–related information-seeking would be positively associated with peritraumatic distress, using media connection and support-seeking, and TSR symptoms. Additionally, we hypothesized that peritraumatic distress will be positively associated with TSR symptoms, while support-seeking behaviors will be negatively associated with TSR symptoms (see Figure 1).

## Methods

### Sample and Recruitment

A convenience sample of 5913 adults from Canada, France, Italy, the United States, and China took part in a web-based survey in April/May 2020 on the psychosocial effects of the COVID-19 pandemic. Participants were recruited through the *snowball technique*. Email invitations were sent to various professional and student associations and to individuals, and an advertisement was shared on social media (ie, Facebook and Twitter).

### Survey and Procedure

The survey was hosted on *SurveyMonkey*. Potential participants were reached through e-mails or social media platforms and were invited to click on the online survey link. Participants were next directed to an online consent form. Before participating in the survey (in one sitting), participants were consented and confirmed that they were 18 years or older. The survey completion rate was very high (n=5913, 92%) among those who clicked on the survey hyperlink. The proportion who received the survey but did not click on the hyperlink remains unknown, however.

### Ethics Approval

Ethics approval was obtained from the Douglas Mental Health University Institute (IUSMD-20-13).

### Measures

#### Media Use

Frequency of media use was assessed with two self-report questions rated on a 5-point scale ranging from 0 (never) to 4 (very often), as similarly done by others [7] in the context of the COVID-19 pandemic [32]: “I maintain closeness and receive the support that I need through social networks and messaging apps” (support-seeking item), and “I looked for and shared information and news on COVID-19 on traditional media, on the internet, or on social networks” (information-seeking item).

#### TSR Symptoms

Severity of TSR symptoms over the previous 7 days was measured using the abridged (6-item) self-report Impact of Event Scale–Revised (IES-6) [33]. The IES-6 measures the severity of intrusions, avoidance, and hypervigilance in response to a stressor or a traumatic event. Items are rated on a 5-point scale, ranging from 0 (not at all) to 4 (extremely), and higher scores (sum of all items; range 0-24) indicate greater symptom severity. A cutoff score of 10 was found to identify clinically significant levels of symptoms [33]. This measure was selected for its comparative performance with the 22-item original scale (*r*=0.95) [33].

#### Peritraumatic Distress

Peritraumatic distress was assessed using the 13-item Peritraumatic Distress Inventory (PDI) [34]. The PDI self-report measures the level of perceived life threat, as well as fear, helplessness, and horror that can occur during and immediately after exposure to a stressor or a traumatic event. Items are rated on a 5-point scale ranging from 0 (not at all) to 4 (extremely true). Higher scores (sum of all items; range 0-52) indicate greater levels of distress. A score of 13 or above indicates clinically significant distress experienced during or shortly after a negative life event [35].

#### COVID-19 Stressors

Exposure to COVID-19–related stressors was measured using a set of 19 dichotomous (1=yes and 0=no) items developed by the team that mapped onto previously used similar questionnaires [31,36]. Specific items included stressors related to the illness itself (eg, have you been diagnosed with COVID-19?), the risk of illness (eg, are you part of an at-risk group?), and other stressors (eg, have you lost your job?). A severity score was calculated by summing all items, resulting in a score ranging between 0 and 19, with higher scores indicating a greater exposure to COVID-19–related stressors.

### Statistical Analyses

All analyses were performed using SPSS (version 27; IBM Corp) and AMOS (version 27; IBM Corp) except for the missing data imputation. A path analysis using a specification search was performed to identify the best fit for the model illustrated in Figure 1. The maximum likelihood method was used to estimate the parameters of the model. Multiple fit indexes were used to evaluate model fit [37]: the chi-square test of absolute fit, the comparative index fit (CFI), the root mean square error of approximation (RMSEA), and the standardized root mean square residual (SRMR).

#### Missing Data

A total of 6409 respondents opened the survey link, from which 496 were removed from the database for not taking part in the study or not responding to any survey question. This led to a final sample size of 5913, in which 65% of the variables had less than 5% of missing values. According to Little’s [38] missing completely at random test (χ^2^_1236_=2135.8; *P*<.001), data were not missing completely at random. Therefore, missing data was imputed using the k-nearest neighbor imputation method with *k*=5 using the VIM package for R (R Foundation for Statistical Computing) [39].

## Results

### Sample

The sample was composed of 5913 adults, most of whom identified as female (n=4681, 79.2%) and resided in Canada (n=1946, 32.9%), the United States (n=1302, 22%), Italy (n=1094, 18.5%), France (n=1036, 17.5%), and China (n=336, 5.7%). Almost half of the sample were essential workers (n=2717, 45.9%), the rest being nonessential workers (n=1578, 26.7%), stay-at-home occupations (ie, students, unemployed, and retired; n=676, 11.4%), and other uncategorized occupations (n=942, 15.9%). Additionally, the sample was, on average, aged 42 (SD 15.24) years. Table 1 presents the sociodemographic information of the sample.

**Table 1:** Sociodemographic, media, and clinical variables.

Sociodemographic, media, and clinical characteristics		Participants (N=5913)
**Marital status, n (%)**		
	Single	1391 (23.52)
	Dating/cohabiting/married	4043 (68.37)
	Separated/divorced/widowed	479 (8.10)
**Ethnicity, n (%)**		
	First Nations	142 (2.40)
	Caucasian	4306 (72.82)
	Black	68 (1.15)
	Latino	316 (5.34)
	Asian	617 (10.43)
	Mixed	123 (2.08)
	Other	341 (5.77)
**Education, n (%)**		
	Preuniversity	837 (14.16)
	Undergraduate level	2064 (34.91)
	Graduate level	3012 (50.94)
**Media variables, mean (SD)**		
	Support-seeking media use	2.88 (1.03)
	Information-seeking media use	2.65 (1.10)
**Clinical variables, mean (SD)**		
	Trauma- and stress-related symptoms^a^	11.24 (5.85)
	Peritraumatic distress^b^	17.53 (10.56)

^a^Abridged 6-item Impact of Event Scale–Revised. A score of 10 or more denotes the presence of clinically significant symptoms [33].

^b^Peritraumatic Distress Inventory. A score of 13 or more is indicative of clinically significant symptoms [35].

### Path Analysis: COVID-19–Related Stressors, Media Use, and TSR Symptoms

The path model was specified as hypothesized in Figure 1. Exposure to COVID-19 stressors and frequency of media use for COVID-19–related information (media information-seeking) were entered as predictors. Peritraumatic distress and frequency of media use for support and connection (media support-seeking) were entered as the intermediary variables, and TSR symptoms were entered as the outcome. As the specified path model was saturated (*df*=0), the nonsignificant direct association between COVID-19 stressors and media support-seeking (β=.004, SE 0.006; CR=0.58; *P*=.57) was removed from the final model. All endogenous variables in the model were ordered categorical with four or more categories. The multivariate kurtosis score of 9.94 suggested that the variables in the final model violated assumptions of multivariate normality [40]. Therefore, estimates and SEs were recalculated using bias-corrected machine learning–bootstrapping (5000 bootstrapped resamples; see Table 2). The lowest percentage of variance explained by the final model was for media support-seeking (*R*^2^=0.09), while the highest percentage of variance explained was for TSR symptoms (*R*^2^=0.57); the model explained 11% of the variance in peritraumatic distress. The final model demonstrated good to excellent indexes of fit (χ^2^_1_=0.3; *P*=.57; CFI=1; RMSEA<0.001; SRMR=0.002) [41,42].

Results from the path analysis revealed that COVID-19 stressors and media information-seeking were significantly and positively associated with peritraumatic distress (*P*_COVID-19 stressors_<.001; *P*_seeking information_<.001). TSR symptoms were weakly but positively associated with COVID-19 stressors (*P*<.001) and media information-seeking (*P*<.001), while peritraumatic distress was strongly and positively associated with TSR symptoms (*P*<.001). Media information-seeking (*P*<.001) and peritraumatic distress (*P*<.001) were both positively associated with media support-seeking. In contrast with our predictions, media support-seeking was significantly and positively associated with TSR symptoms (*P*=.006), although the effect was very small. Standardized parameter estimates of the direct associations can be found in Figure 1, and the unstandardized parameters estimates can be found in Table 2.

**Figure 2:**
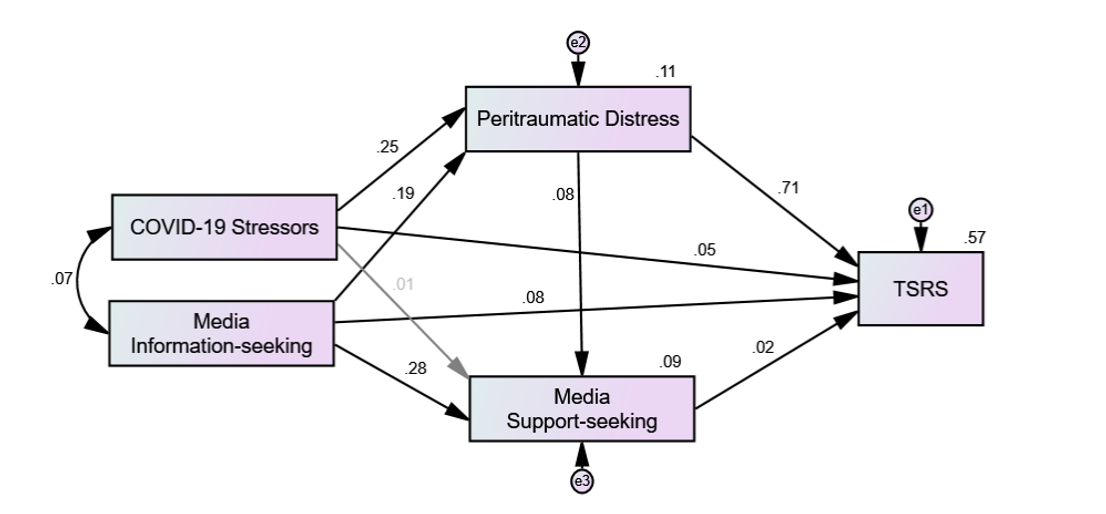
Path analysis and results. TSRS: trauma- and stressor-related symptoms.

Black lines represent statistically significant associations, while the gray ones represent nonsignificant associations that were removed from the final model. Numbers above the lines represent standardized beta coefficients, which were used to interpret the strength of the associations. The explained variance (*R*^2^) is indicated on the upper-right corner of the intermediary and outcomes variables. Error terms are represented by the e-labelled circles above the intermediary and outcome variables (Figure 2).

**Table 2:** Standardized parameter estimates of direct effects.

Independent variables	Dependent variables	Noncorrected estimates, B (SE)	Bias-corrected estimates		
			B (SE)	95% CI	*P* value
COVID-19 stressors	Peritraumatic distress	1.24 (0.06)	1.24 (0.08)	1.10-1.39	<.001
Media information	Peritraumatic distress	1.78 (0.12)	1.79 (0.12)	1.55-2.01	<.001
Peritraumatic distress	Media support	0.01 (<0.01)	0.01 (<0.01)	0.01-0.01	<.001
Media information	Media support	0.26 (0.01)	0.26 (0.01)	0.23-0.29	<.001
Media information	Distress	0.45 (0.05)	0.45 (0.05)	0.35-0.54	<.001
Media support	Distress	0.14 (0.05)	0.14 (0.05)	0.03-0.24	.008
Peritraumatic distress	Distress	0.39 (0.01)	0.39 (0.01)	0.38-0.40	<.001
COVID-19 stressors	Distress	0.14 (0.02)	0.14 (0.02)	0.09-0.18	<.001

### Analysis of Indirect Associations

The path analysis revealed that the associations between the independent variables (exposure to COVID-19–related stressors and media information-seeking) and the outcome variable (TSR symptoms) passed through levels of peritraumatic distress and media support-seeking. As per Table 3, both peritraumatic distress and support-seeking were intermediary variables, as they were both significantly correlated with at least one independent variable and the outcome [43].

As recommended by Preacher and Hayes [44], the 95% bias-corrected bootstrap CIs were computed to examine whether the indirect associations of the predictors on the outcome were significantly different from zero when using 5000 bootstrapped samples. The bootstrapped indirect associations of exposure to COVID-19 stressors and media information-seeking through peritraumatic distress on TSR symptoms were statistically significant (β_COVID-19 stressors_=.49, 95% CI 0.43-0.55; β_seeking information_=.70, 95% CI 0.61-0.79). Additionally, the indirect associations of media information-seeking through media support-seeking on TSR symptoms was also statistically significant (β=.04, 95% CI 0.01-0.06), although this association was negligible.

**Table 3:** Pearson correlation coefficients between variables included in the path analysis (N=5913)^a^.

Variables	COVID^b^	IM^c^	SM^d^	PDI^e^	TSR^f^
COVID	—^g^				
IM	0.07	—			
SM	0.05	0.30	—		
PDI	0.28	0.20	0.14	—	
TSR	0.24	0.24	0.15	0.75	—

^a^*P*<.001 for all correlation coefficients. The correlation is significant at the .001 level.

^b^COVID: COVID-19–related stressors.

^c^IM: information-seeking media use.

^d^SM: support-seeking media use.

^e^PDI: peritraumatic distress.

^f^TSR: trauma- and stress-related symptoms.

^g^Not applicable.

## Discussion

### Media Use and Trauma- and Stressor-Related Symptoms

The goal of this study was to understand the combined associations between exposure to COVID-19–related stressors and media use for COVID-19–related information on the development of TSR symptoms while considering the role that peritraumatic distress and media use for support-seeking might play. In line with our main hypothesis, exposure to COVID-19 stressors and media use for obtaining COVID-19–related information led to elevated peritraumatic distress that, in turn, was associated with higher TSR symptoms. However, contrary to predictions, media use for support and connection did not appear to be related with the association between exposure to COVID-19 stressors and TSR symptoms or between the use of media for obtaining COVID-19–related information and TSR symptoms. In fact, the association between media use for support and TSR symptoms was positive, rather than negative, suggesting that using social media for support, alone, may not be sufficient to protect against the mental health effects of exposure to the pandemic-related stressors. However, such an association may also be attributable to endogeneity issues, which can occur when observed associations are due to correlations between the model and error terms rather than the variables of interest [45]. Further longitudinal research is, therefore, necessary to better understand the associations and potential causality links between media use for support-seeking, peritraumatic distress, and TSR symptoms.

### Peritraumatic Distress, Media Use, and Trauma- and Stressor-Related Symptoms

Peritraumatic distress was strongly associated not only with severity of TSR symptoms but also with exposure to COVID-19 stressors and seeking information through media on trauma-related symptoms. Such findings are consistent with prior literature, as the predictive power of peritraumatic distress on the later development and severity of TSR symptoms and, more precisely, of PTSD is well documented [25,46]. Moreover, in this study, both exposure to COVID-19–related stressors and media use for seeking COVID-19–related information were significantly and similarly associated with peritraumatic distress. Although this was slightly stronger for exposure to COVID-19 stressors, these results suggest that the frequency with which individuals consume COVID-19–related information through the media may be related with significant peritraumatic distress reactions, which in turn, can increase the severity of TSR symptoms. This is consistent with current literature suggesting that media exposure during the pandemic is associated with increased anxiety, depression, and secondary trauma symptoms across various populations [47-50]. Additionally, a statistically significant association was found between peritraumatic distress and media use for seeking support, suggesting that individuals who are more distressed may seek more support through social media. Further investigation of this issue is warranted, as it is not possible to verify the direction of the association with the current data.

Contrary to predictions, findings from this study suggest that media use for support and connection may not be sufficient to reduce the pathogenic effects of the pandemic. Considering that TSR symptoms result from exposure to environmental stressors, it is possible that individuals who frequently use media to actively seek support and connection during the pandemic are simultaneously exposed to overwhelming information about the pandemic [17], resulting in an increased risk of TSR symptoms. Importantly, however, the examination of different mental health outcomes may yield different results. For instance, some studies have suggested that, during the pandemic, individuals who sought support through social media reported lower levels of loneliness [51,52], while others have found that this behavior may not negatively affect levels of anxiety and mental health [18]. Thus, future studies may incorporate additional measures of mental health in the examination of the association between media use for support-seeking and mental health.

### Limitations and Future Directions

The cross-sectional nature of the data prevents making causal inferences. For instance, the small association between support-seeking through media and TSR symptoms may be partly explained by the level of distress experienced by individuals [53]. Additionally, the media variables used in this study were constructed of single items, which may have affected the stability of the model. However, the large sample size and variability in the data allowed testing of a strong model and, consequently, increased confidence in the results. Importantly, though, given that the sample is composed of a higher proportion of female respondents, caution must be considered when generalizing the results. In this study, TSR symptoms were measured using a self-reported questionnaire, which may yield a somewhat higher symptom severity (eg, [54]). Further investigations will need to address the effect of media use on TSR symptoms longitudinally to understand this association overtime while also evaluating TSR symptoms using a clinician-administered measure. Finally, given that media use may affect other indicators of mental health, such as anxiety or depression, future research may wish to include additional mental health outcomes.

This paper highlights important considerations from both empirical and societal perspectives. First, future research should explore the effects of media on mental health according to how it is used, as results from this study emphasize the differences between the use of media to seek information and support on TSR symptoms. Second, considering the recommendations stemming from national and international guidelines [1-3], there is a need to inform the population on how the use of media impacts various aspects of mental health. Although it is important to remain connected and informed during these unprecedented times, too much use of traditional and social media may negatively impact mental health. Educating the public on how to use media in a crisis to remain informed without jeopardizing their mental health should be prioritized, as this could help prevent detrimental mental health consequences such as TSR symptoms.

## Abbreviations

CFI: comparative index fit
DSMM: differential susceptibility to media effects model
IES-6: Impact of Event Scale–Revised
PDI: Peritraumatic Distress Inventory
PTSD: posttraumatic stress disorder
RMSEA: root mean square error of approximation
SRMR: standardized root mean square residual
TSR: trauma- and stressor-related

## Data Availability

Data access can be granted upon request.
